# Allogeneic umbilical cord blood-derived mesenchymal stem cell implantation versus microdrilling combined with high tibial osteotomy for cartilage regeneration

**DOI:** 10.1038/s41598-024-53598-9

**Published:** 2024-02-09

**Authors:** Se-Han Jung, Bum-Joon Nam, Chong-Hyuk Choi, Sungjun Kim, Min Jung, Kwangho Chung, Jisoo Park, Youngsu Jung, Sung-Hwan Kim

**Affiliations:** 1 Arthroscopy and Joint Research Institute, Yonsei University College of Medicine, Seoul, Republic of Korea; 2 Department of Orthopedic Surgery, Gangnam Severance Hospital, Yonsei University College of Medicine, 211 Eonju-ro, Gangnam-gu, Seoul, 06273 Republic of Korea; 3 Department of Orthopedic Surgery, Severance Hospital, Yonsei University College of Medicine, Seoul, Republic of Korea; 4 Department of Radiology, Gangnam Severance Hospital, Yonsei University College of Medicine, Seoul, Republic of Korea; 5 Department of Orthopedic Surgery, Yongin Severance Hospital, Yonsei University College of Medicine, Yongin, Republic of Korea

**Keywords:** Stem cells, Mesenchymal stem cells, Regeneration, Osteoarthritis

## Abstract

This study compared cartilage regeneration outcomes in knee osteoarthritis (OA) using allogeneic human umbilical cord blood-derived mesenchymal stem cell (hUCB-MSC) implantation and microdrilling with high tibial osteotomy (HTO). Fifty-four patients (60 knees) were included: 24 (27 knees) in the hUCB-MSC group and 30 (33 knees) in the microdrilling group. Both groups showed significant improvements in pain and functional scores at 6, 12, and 24 months compared to baseline. At 24 months, the hUCB-MSC group had significantly improved scores. Arthroscopic assessment at 12 months revealed better cartilage healing in the hUCB-MSC group. In subgroup analysis according to the defect site, hUCB-MSC implantation showed superior cartilage healing for anterior lesions. In conclusion, both treatments demonstrated effectiveness for medial OA. However, hUCB-MSC implantation had better patient-reported outcomes and cartilage regeneration than microdrilling. The study suggests promising approaches for cartilage restoration in large knee defects due to OA.

## Introduction

Osteoarthritis (OA) is one of the common causes of knee pain. Without appropriate treatment, this condition tends to progress, owing to limited capacity for natural healing^1–3^. Varus deformities combined with cartilage defects on the medial femoral condyle (MFC) are one of the major underlying pathologies leading to a significant increase in mechanical loads in the medial compartment^4,5^. Biomechanical studies have demonstrated that high tibial osteotomy (HTO) can reduce loads in the medial compartment, subsequently reducing the peak pressure to the associated focal cartilage defects^6–8^. Due to these biomechanical effects, HTO alone offers excellent short- and mid-term outcomes, serving as an effective treatment option for medial compartment OA with varus deformity; however, these outcomes tend to deteriorate over time^9–12^. Long-term survivals after HTO were reported to be 64–93.2% at 10 years, and 46–85.1% at 20 years, suggesting the possibility of future total knee arthroplasty conversion for relatively young patients^13^. It has been reported that cartilage defects can be partially or entirely covered by the regenerated cartilage after HTO even without cartilage regeneration procedures^14,15^. However, the possibility of full coverage of the MFC defects is unsatisfactory, and tissue quality of the regenerated cartilage (fibrous cartilage) is questionable. To overcome these limitations and improve longevity and long-term surgical outcomes after HTO, additional cartilage regenerative procedures are increasingly being combined.

Cartilage regeneration procedures, such as microfracture (MFx), microdrilling, and autologous chondrocyte implantation (ACI) with concomitant HTO, may improve regenerated cartilage volume and quality, which can be possibly related to long-term outcomes^16–18^. The MFx technique is applicable for the repair of small- to mid-sized cartilage defects (< 4–5 cm^2^), especially for focal and contained defects^19,20^. However, the results for larger defects with arthritic change are suboptimal, and cartilage tends to deteriorate within a few years^21–23^. Moreover, several studies have indicated no additional improvement in clinical outcomes when MFx was added to HTO^24,25^. ACI is not routinely recommended in older patients because of accelerated cellular senescence and the decreased reparative potentials of autologous cells^26^.

Recently, mesenchymal stem cells (MSCs) with concomitant HTO have been proposed as a potential treatment option for cartilage restoration in older patients^27–29^. MSCs can be obtained from various tissues of the human body such as the bone marrow (BM), synovium, adipose tissue, and umbilical cord^30^. Among the variously sourced MSCs, human umbilical cord blood-derived MSCs (hUCB-MSCs) have advantages of non-invansive cell collection, high capacity for expansion, and low immunogenicity for therapeutic applications as an off-the-shelf allogeneic product^31–33^. Implantation of hUCB-MSCs demonstrated both safety and efficacy in cartilage repair for older patients with knee OA with no reported serious adverse events^34,35^. Its allogeneic use allows for one-stage surgeries without additional autologous tissue harvests. In this regard, hUCB-MSCs minimizes the burden on patients, especially when used with HTO, providing an advantageous option for those seeking optimal results.

This study aimed to compare clinical and radiographic outcomes, as well as second-look arthroscopic outcomes, after combining HTO with hUCB-MSC or microdrilling treatments. Further, it aimed to determine the relationship between articular cartilage regeneration and the defect site. Based on second-look arthroscopy, we hypothesized that HTO with hUCB-MSC implantation would demonstrate better clinical outcomes and superior articular cartilage regeneration than HTO combined with microdrilling.

## Methods

### Patients

We retrospectively reviewed the medical records of patients who underwent implantation of allogenic hUCB-MSCs or microdrilling with concomitant HTO for the treatment of medial compartmental OA at one hospital between April 2019 and May 2021. Study design was approved by the Institutional Review Board and Ethics Committee (Gangnam Severance Hospital, Institutional Review Board). All experiments were performed in accordance with relevant guidelines and regulations. This study received exemption from informed consent by the Institutional Review Board and Ethics Committee. We included patients who underwent plate removal and second-look arthroscopy to check cartilage regeneration after complete bony union at least at the 1-year follow-up and had a near full-thickness cartilage defect in the MFC (International Cartilage Repair Society [ICRS] grade 3 or 4) with varus deformity.

We excluded patients with grade IV OA of the medial compartment (identified by radiological assessment according to the Kellgren and Lawrence system)^36^, knee range of motion < 100° with flexion contracture > 15, additional surgical procedures of the same knee, knee ligament injuries, metabolic arthritis, joint infections, articular cartilage lesions in the lateral compartment, or a follow-up duration of less than 1 year.

### Cell Preparation

Allogeneic hUCB-MSCs were produced at a cell manufacturing facility operated by MEDIPOST Co. Ltd. (Seongnam-si, Gyeonggi-do, South Korea) in full compliance with the Good Manufacturing Practice requirements of the Ministry of Food and Drug Safety, as well as with donor screening, cell isolation and expansion, and quality control measures. The therapeutic use of this cell product for cartilage repair was reviewed and approved by the Korea Food and Drug Administration in January 2012. Safety was assessed by a previous clinical trial by Park et al^34^., and no serious adverse events were reported. Commercially available hUCB-MSCs (Cartistem®, Medipost Inc., a composite of hUCB-MSCs 0.5 × 10^7^/ml and freeze-drying sodium hyaluronate [HA]) were mixed to a gel-type consistency according to the manufacturer’s instructions prior to the application during surgery^34^.

### Surgical techniques and postoperative management

All procedures were performed under general or spinal anesthesia. Diagnostic arthroscopy was performed before the osteotomy procedure, and the status of the articular cartilage was evaluated thoroughly. After a complete inspection of the joints and assessment of cartilage defects, biplane medial open-wedge HTO was performed in the same manner as in the previous study^37^.

**Figure 1:**
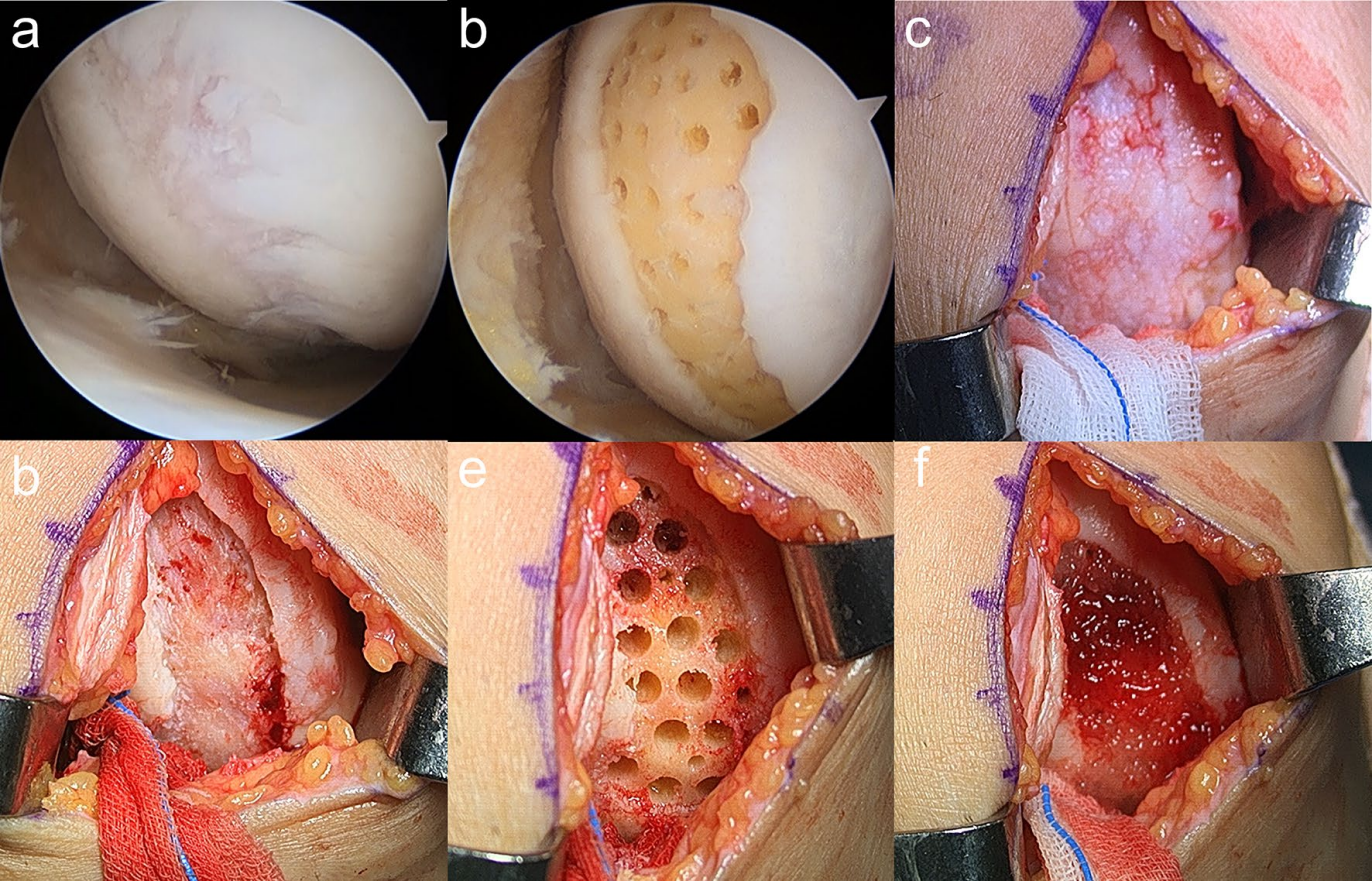
hUCB-MSC implantation and arthroscopic microdrilling procedures. (**a**) Arthroscopic view of the cartilage defect on medial femoral condyle viewed from the standard anterolateral portal in microdrilling group. (**b**) After arthroscopic microdrilling. (**c**) Exposure of the large cartilage defect on medial femoral condyle through mini-open arthrotomy. (**d**) A large cartilage defect extending anterior to posterior. (**e**) Drilling of the cartilage defect. (**f**) Implantation of the hUCB-MSCs.

In the microdrilling group, the chondral defect lesion was debrided and prepared using gouges and curettes prior to the HTO procedure. Multiple drill holes (1.5 × 14 mm [diameter × depth]; approximately 1–2 mm apart) were then made in the subchondral bone (Fig. 1a, b). In the hUCB-MSCs group, a mini-arthrotomy through an incision of approximately 3–4 cm in length was made after the HTO procedure (Fig. 1c). After preparing the chondral lesion, multiple drill holes of two different sizes (4 × 7 mm, 2 × 7 mm [diameter × depth], approximately 2 mm apart) were made in the subchondral bone for the temporary containment of the hUCB-MSC-HA mixture and the marrow stimulation (Fig. 1d, e). After preparing the lesion, the hUCB-MSC and HA mixture was slowly implanted into all the drill holes, and the defect area was subsequently covered completely with the mixture (Fig. 1f). No additional scaffolds or procedures were applied for sealing, based on the guidance by which this practice was developed and approved. The wound was closed, and a long leg splint was applied.

After the procedure, knee motion was restricted using a hinged knee brace during daily activities for a total of 10 weeks. Continuous passive range of motion exercises were recommended immediately after surgery, starting at 60 degrees and increasing by 30 degrees every two weeks, with the goal of achieving 120 degrees to full range of motion by six weeks postoperatively. Weight-bearing was restricted for a total of 10 weeks using crutches. Toe-touch weight-bearing was permitted for the initial four weeks, followed by six weeks of partial weight-bearing, allowing for approximately 50% of the normal load during walking.

### Clinical evaluation

To assess pain and function of the knees, we used the visual analog scale (VAS), International Knee Documentation Committee (IKDC) subjective score, and Lysholm Knee Scoring Scale preoperatively as well as at 6, 12, and 24 months after surgery^38,39^.

### Magnetic Resonance Imaging evaluation

The quality of cartilage repair tissue, which was the primary efficacy endpoint, was evaluated via magnetic resonance imaging (MRI) using a 3-T scanner preoperatively and at 12 months postoperatively. MRI images were analyzed using the Magnetic Resonance Observation of Cartilage Repair Tissue (MOCART) 2.0 Knee Score. Although MRI cannot accurately determine the status of cartilage repair^40^, the MOCART 2.0 score (0 = worst cartilage status, 100 = best articular cartilage status) was highly correlated with clinical outcome^41,42^. To avoid bias, two orthopedic surgeons specialized in knee surgery and one radiologist trained in musculoskeletal radiology evaluated the MR images acquired from all participants in a blinded manner. Each of the scores for the variables of the MOCART score reported by the two orthopedic surgeons and radiologist were recorded separately, and the total MOCART score was calculated from the mean of the three scores.

### Arthroscopic evaluation

Second-look arthroscopy was performed approximately one year after the initial HTO surgery in patients who underwent surgical treatment for hardware removal. During the second-look procedure, the repaired cartilage was inspected and evaluated using the ICRS cartilage repair assessment (CRA) scoring system (score 0–12), including the degree of defect fill, the degree of graft integration to the adjacent normal articular surface, and the gross appearance of the graft surface^43^.

### Subgroup analyses according to the defect location

The MOCART and ICRS CRA scores were used to evaluate the defect site according to the following criteria. The distal medial femoral condyle is divided into four sub-regions: trochlea, anterior, middle, and posterior femur.

**Figure 2:**
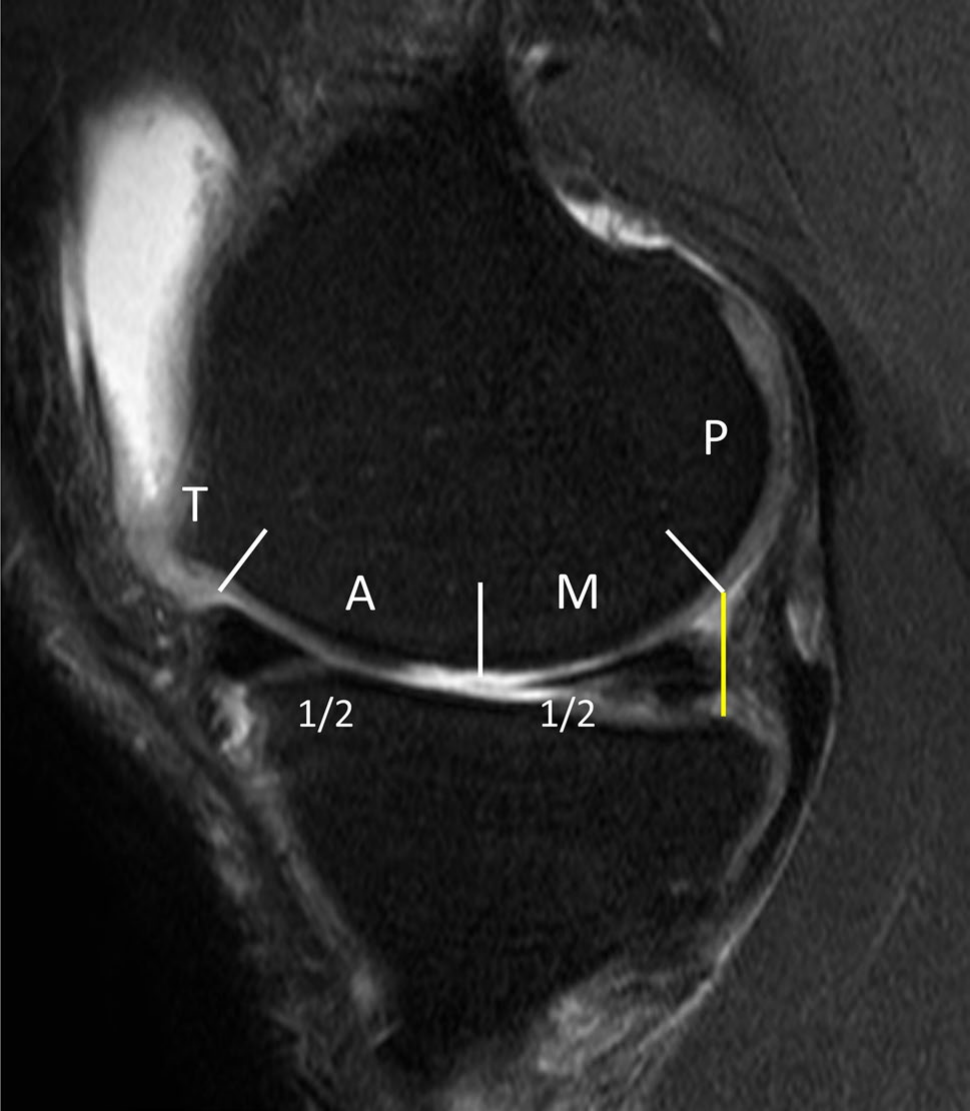
Anatomical subdivision of the medial femoral condyle into trochlea (T), anterior (A), middle (M), and posterior (P) regions on sagittal projection.

*Method of dividing subregions* (Fig. 2).

To divide the trochlea and anterior regions, on the sagittal image, terminal sulcus of femoral condyles was used, which matches the margin of the anterior horn of the meniscus in full extension. The division between the middle and posterior femur is a line constructed tangentially to the posterior edge of the tibial articular surface. Reference starting point of the posterior region was the tibial articular surface, not the meniscus posterior horn since most of the patients showed meniscal extrusion of medial meniscus due to underlying osteoarthritis. The area between the trochlea and the posterior area was then equally divided into the anterior and middle regions. This subdivision was carried out based on the preoperative MRIs. Maintaining the established subdivisions, cartilage regeneration within these subregions was then assessed using each MOCART 2.0 scores on postoperative MRIs.

### Statistical analysis

Differences in normally distributed variables, such as clinical outcome, MOCART 2.0, and ICRS CRA between hUCB-MSC implantation and microdrilling, were analyzed using paired and independent *t*-tests. The Wilcoxon signed-rank test and Mann–Whitney U test were used to analyze differences when normality was absent. A repeated-measures analysis of variance test was performed for the subgroup analysis (the relationship between articular cartilage regeneration and defect location), and Bonferroni correction was performed for post hoc analysis. The level of significance was set at *P* < 0.05. All statistical analyses were performed using the IBM SPSS Statistics (version 26.0; IBM, Armonk, New York, USA). Statistical power analysis was performed using G*power version 3.1 (University Düsseldorf, Düsseldorf, Germany). Using the significance level (alpha) of 0.05, statistical power (1-beta) of the independent *t*-tests between the two groups was 84.4% for VAS score, 83.5% for IKDC score, and 66.8% for ICRS CRA scores. For tests examining statistical differences in MOCART and ICRS CRA scores based on each defect location, statistical power was over 99% for the statistically significant data in the hUCB-MSC group. In the microdrilling group, statistical power of comparison between middle and posterior locations was relatively low (34–59%), but comparison between anterior and other locations had relatively high statistical power (58.2%, 88–99%).

### Institutional review board (IRB)

This study was approved by the institutional review board of our institution (2022 1107 001).

## Results

### Demographics

**Figure 3:**
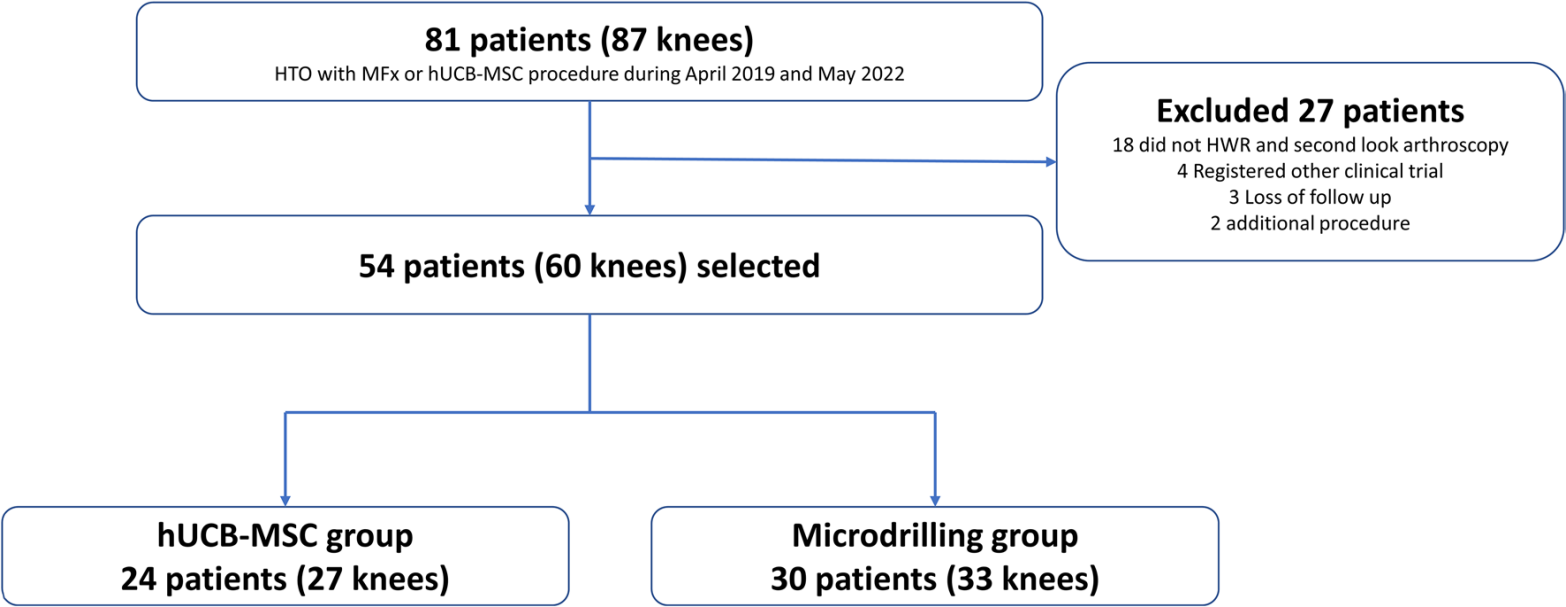
Flowchart of patient inclusion in the study.

Of the 81 eligible patients (87 knees), 54 patients who met the inclusion criterion were included in this study and divided into two groups according to cartilage procedure. The distribution of patients was as follows: (1) group 1, 24 patients (27 knees) with implantation of allogenic hUCB-MSCs, (2) group 2, 30 patients (33 knees) microdrilling with concomitant HTO (Fig. 3). The mean age of participants was 56.88 years in the hUCB-MSC group and 59.91 years in the microdrilling group; mean body mass index was 26.55 (hUCB-MSC) and 26.60 (microdrilling). The mean lesion size of MFC and trochlear (TCH) defects was 7.25 and 2.17 cm^2^ in the hUCB-MSC group, and 6.61 and 2.48 cm^2^ in the microdrilling group, respectively. Besides the time to hardware removal, the baseline characteristics were similar between the two groups (Table 1). Since the clinical trial was conducted on the hUCB-MSC group, stricter follow-up was possible, and the period until hardware removal was short.

**Table 1:** Comparison of baseline characteristics.

Parameters	hUCB-MSC (*n* = 27)	Microdrilling (*n* = 33)	*P* value
Side (R/L)	13/14	16/17	0.98
Sex (M/F)	7/20	11/22	0.34
Age (yr)	56.88 ± 6.97	59.91 ± 4.56	0.06
Body mass index (kg/m^2^)	26.55 ± 3.47	26.60 ± 3.68	0.95
Follow-up period (months)	22.07 ± 7.61	18.61 ± 6.18	0.56
Defect size (MFC, cm^2^)	7.25 ± 2.51	6.61 ± 3.31	0.42
Defect size (TCH, cm^2^)	2.17 ± 1.31	2.48 ± 1.25	0.58
Time to hardware removal (months)	12.33 ± 0.68	13.00 ± 1.39	**0.027**

*MFC*, medial femoral condyle; *TCH*, trochlear.

Significant values are in [bold].

### Clinical outcomes

**Figure 4:**
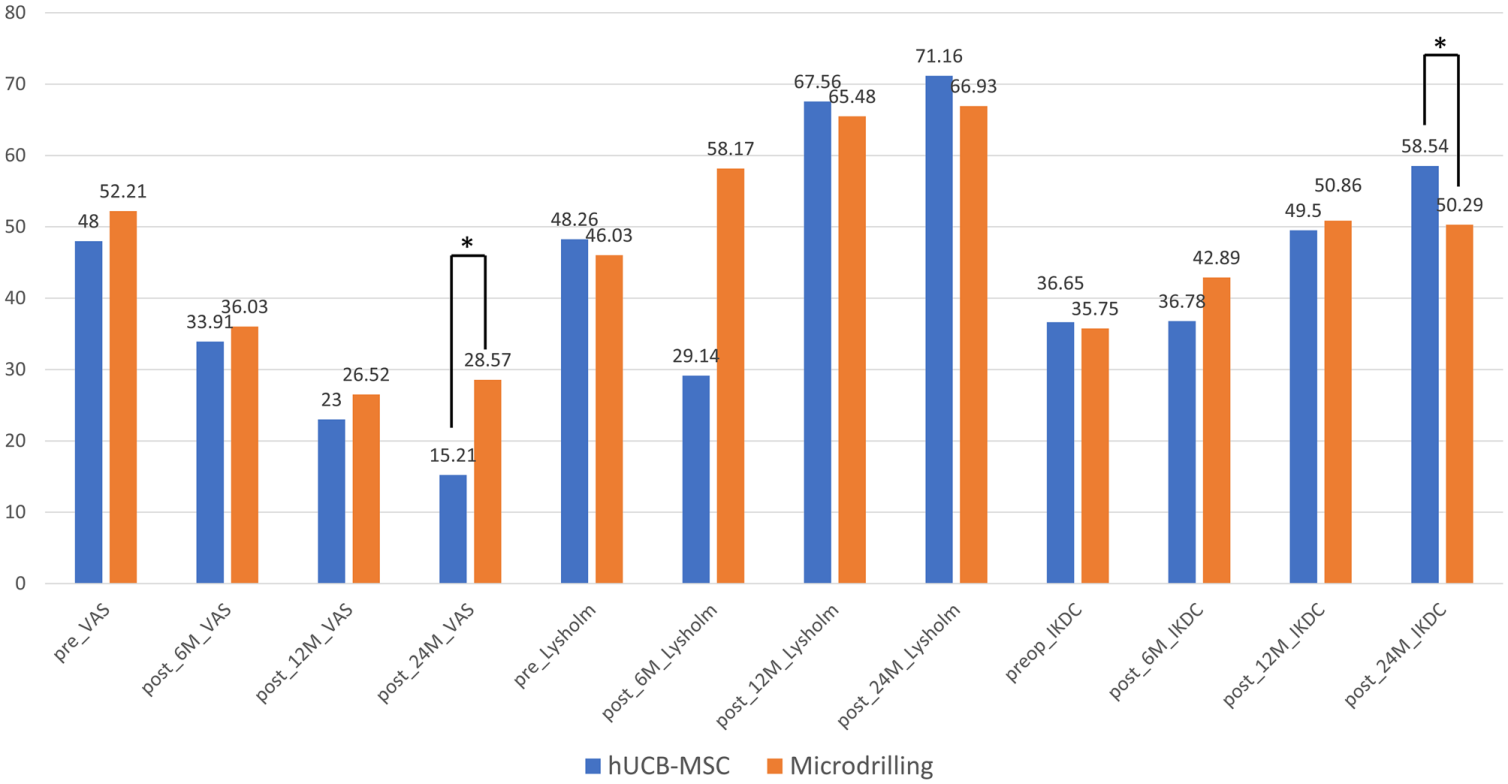
Patient-reported outcome scores across time points between groups. *Significant differences between groups with respect to scoring distributions at a specific time point in the student *t*-test at *α* = .05.

No significant differences in the preoperative clinical scores were observed between the two groups. From the preoperative to the final follow-up, significant improvements regarding all clinical scores (VAS pain, Lysholm, IKDC scores) were observed in both the hUCB-MSC and microdrilling groups without serious adverse events (all *P* < 0.001). At 24 months after surgery, the hUCB-MSC group demonstrated significantly better clinical scores than the microdrilling group on both VAS (15.21 vs 28.57, *P* = 0.016) and IKDC scores (58.54 vs 50.29, *P* = 0.038) (Table 2 and Fig. 4).

**Table 2:** Patient-reported outcomes at each time points (0, 6 months, 12 months, 24 months).

	hUCB-MSC	Microdrilling	*P* value
Pre_VAS	48 ± 25.1	52.21 ± 27.46	0.54
Post_6M_VAS	33.91 ± 18.86	36.03 ± 25.18	0.74
Post_12M_VAS	23 ± 14.08	26.52 ± 18.42	0.41
Post_24M_VAS	15.21 ± 13.12	28.57 ± 20.23	**0.016**
Pre_Lysholm	48.26 ± 16.89	46.03 ± 18.96	0.64
Post_6M_ Lysholm	29.14 ± 20.32	58.17 ± 20.79	0.13
Post_12M_ Lysholm	67.56 ± 13.34	65.48 ± 20.93	0.66
Post_24M_ Lysholm	71.16 ± 20.37	66.93 ± 14.11	0.51
Pre_IKDC	36.65 ± 13.74	35.75 ± 17.12	0.83
Post_6M_ IKDC	36.78 ± 9.30	42.89 ± 14.16	0.083
Post_12M_ IKDC	49.5 ± 11.23	50.86 ± 13.23	0.6
Post_24M_ IKDC	58.54 ± 11.72	50.29 ± 9.46	**0.038**

hUCB-MSC, human umbilical cord blood-derived mesenchymal stem cell; Pre, preoperative; *VAS*, visual analog scale; Post, postoperative; *IKDC*, International Knee Documentation Committee.

Significant values are in [bold].

### Radiologic and arthroscopic outcomes (Fig. 5)

**Figure 5:**
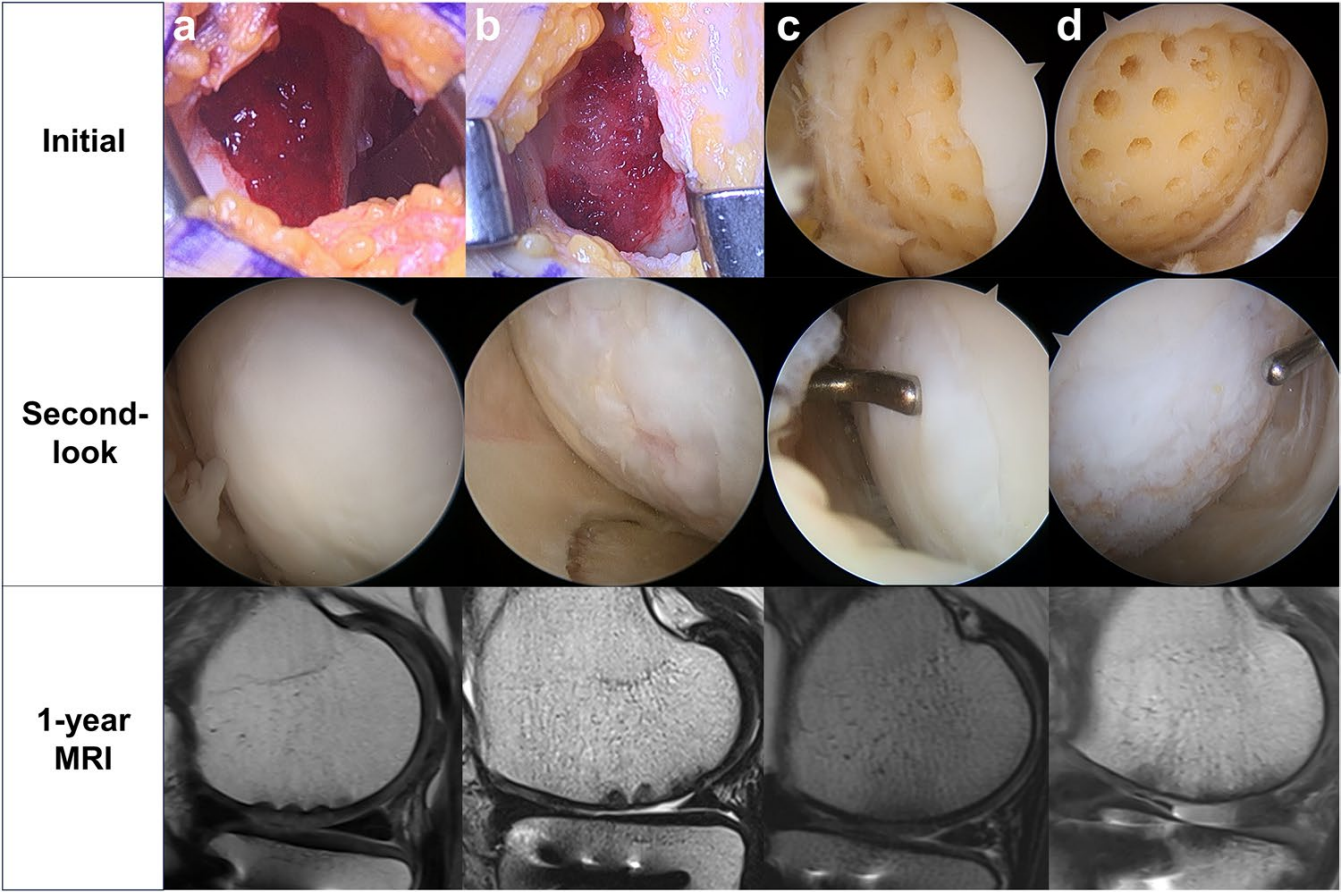
Cartilage regeneration outcomes in patients underwent two different procedures, hUCB-MSC implantation (a, b) and arthroscopic microdrilling (c, d). Initial, second-look arthroscopy, and postoperative MRI images depict the best (a, c) and the worst (b, d) cases among patients with anterior-to-mid lesions. (**a**) Fully covered medial femoral condyle (MFC) defect with cartilaginous tissue, achieving a MOCART score of 70. (**b**) Incomplete coverage of the MFC defect with irregular cobblestone appearance, resulting in a MOCART score of 35. (**c**) Complete coverage of the MFC defect after arthroscopic microdrilling, with softness on probing noted. (**d**) Minimal coverage of the MFC defect, presenting as soft and thin tissue, with a MOCART score of 25. MOCART, Magnetic Resonance Observation of Cartilage Repair Tissue.

One year after surgery, 85% (51/60) of the patients underwent MRI. There was no statistically significant difference in MOCART 2.0 scores between the two groups (53.7 ± 9.07 vs 53.04 ± 14.49, *p* = 0.85).

The mean time to second-look arthroscopy was 12.7 months after the initial surgery. The second-look arthroscopic findings related to articular cartilage regeneration according to the ICRS CRA grading system are summarized in Table 3. The hUCB-MSC group showed significantly better cartilage regeneration than the microdrilling group in the ICRS CRA score (9.41 vs 7.94, *p* = 0.021).

**Table 3:** Comparison of MRI and second-look arthroscopy outcomes between the groups.

	hUCB-MSC	Microdrilling	*P* value
MOCART 2.0 score	53.7 ± 9.07	53.04 ± 14.49	0.85
ICRS CRA score	9.41 ± 1.76	7.94 ± 2.78	**0.021**

hUCB-MSC, human umbilical cord blood-derived mesenchymal stem cell; *MOCART*, Magnetic resonance observation of cartilage repair tissue; *ICRS CRA*, International Cartilage Repair Society Cartilage repair assessment.

Significant values are in [bold].

The results from the subgroup comparative analysis of cartilage regeneration depending on defect location (anterior, middle, posterior) are presented in Tables 4 and 5 for both hUCB-MSC and microdrilling groups. In the hUCB-MSC group, the anterior lesion showed significantly higher MOCART (*P* < 0.001) and ICRS CRA (*P* < 0.001) scores compared to the middle and posterior lesions (Table 4). In the microdrilling group, the anterior lesion also showed significantly higher MOCART and ICRS CRA scores compared to the middle and posterior lesions (Table 5). The middle lesion showed higher MOCART (*P* = 0.034) and ICRS CRA (*P* = 0.049) scores than the posterior lesions in the microdrilling group. However, statistical power of comparisons between the middle and posterior lesions was relatively low (34–59%).

**Table 4:** Outcomes of MRI and second-look arthroscopy by the defect locations in the hUCB-MSC implantation group.

hUCB-MSC	Anterior	Middle	Posterior	P^a^ (Ant-Mid)	P^a^ (Mid-Post)	P^a^ (Ant-Post)
MOCART 2.0	69.29 ± 7.77	53.26 ± 12.49	45.00 ± 10.49	** < 0.001**	0.273	** < 0.001**
ICRS CRA	10.72 ± 1.67	7.6 ± 2.76	6.00 ± 2.12	** < 0.001**	1.000	** < 0.001**

^a^ *P* values were corrected using Bonferroni method due to multiple comparison.

MRI, Magnetic resonance imaging; hUCB-MSC, human umbilical cord blood-derived mesenchymal stem cell; MOCART, Magnetic resonance observation of cartilage repair tissue; ICRS CRA, International Cartilage Repair Society Cartilage repair assessment; Ant, anterior; Mid, middle; Post, posterior.

Significant values are in [bold].

**Table 5:** Outcomes of MRI and second-look arthroscopy by the defect locations in the microdrilling group.

Microdrilling	Anterior	Middle	Posterior	P^a^ (Ant-Mid)	P^a^ (Mid-Post)	P^a^ (Ant-Post)
MOCART 2.0	57.83 ± 14.91	51.14 ± 14.22	40.56 ± 13.56	**0.033**	**0.034**	**0.007**
ICRS CRA	9.54 ± 2.74	7.60 ± 3.22	5.8 ± 2.44	**0.003**	**0.049**	**0.002**

^a^ *P* values were corrected using Bonferroni method due to multiple comparison.

MRI, Magnetic resonance imaging; MOCART, Magnetic resonance observation of cartilage repair tissue; ICRS CRA, International Cartilage Repair Society Cartilage repair assessment; Ant, anterior; Mid, middle; Post, posterior.

Significant values are in [bold].

## Discussion

The main aim of this study was to evaluate the effect of hUCB-MSC treatment combined with HTO by conducting comparative analysis of clinical and radiological outcomes with the microdrilling group. These outcomes improved in both groups regardless of the treatment administered. However, the hUCB-MSC procedure was more effective than microdrilling in terms of clinical and cartilage regeneration outcomes.

A number of previous studies comparing hUCB-MSCs and bone marrow aspirate concentrate reported similar improvements in clinical outcomes for both procedures; however, hUCB-MSCs showed better cartilage regeneration^29,44,45^. Another study compared microfractures and hUCB-MSCs and reported significant improvements in clinical outcomes and cartilage regeneration in the hUCB-MSC group^35^. There was also a report that the clinical and radiologic outcomes were improved in patients who underwent the hUCB-MSC procedure together with HTO^46–48^. Similar to these studies, in the current study, hUCB-MSC performed with HTO yielded superior results compared to microdrilling in terms of clinical outcomes, second-look assessment, and MRI findings.

**Figure 6:**
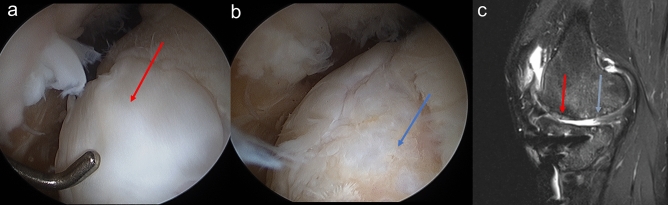
Second-look arthroscopic findings of (**a**) the anterior lesion of MFC (ICRS Grade I) and (**b**) the mid to posterior lesion of MFC. (ICRS Grade IIIb) (**c**) At the 1-year follow-up, the anterior repaired cartilage (red arrow) completely filled the defect, but mid to posterior cartilage (blue arrow) showed an irregular surface and poor integration with incompletely filled defect at T2 PD FS MRI images.

New findings were discovered during MRI analysis and second-look arthroscopy analysis at 1 year postoperatively. Cartilage regeneration status assessed using MOCART and ICRS CRA scores varied depending on the location of the defect on MFC, even within a single patient (Fig. 6). Therefore, a subgroup analysis was conducted. The results are presented in Tables 4 and 5. In the hUCB-MSC group, the anterior lesion showed significantly higher MOCART and ICRS CRA scores as compared to the middle and posterior lesion. In the microdrilling group, there was a statistically significant trend that more anterior lesions showed higher MOCART and ICRS CRA scores compared to the more posterior lesions after cartilage regeneration. However, comparison between the middle and posterior lesions showed relatively low statistical power on further analysis. The results showed that cartilage regeneration in the anterior lesion was superior to that in the posterior lesion in both groups. This subregional difference is thought to be related to meniscal functional loss and loading conditions in OA patients^49,50^. Degenerative meniscal tears occur mainly in the posterior 1/3 of the medial meniscus. In the older adults’ group, it is reported that approximately 80% of articular cartilage lesions are accompanied by this^51^. Moreover, it was reported that the detrimental effects of these meniscal tears do not uniformly affect all regions of the femoral condyles but rather are concentrated in specific areas^49^. In this study, degenerative meniscal tears of the posterior horn of medial meniscus were observed in most patients with some degree of meniscal extrusions, indicating meniscal functional loss. On a subregional MRI analysis of cartilage loss in OA knees by Jørgensen et al^50^., the greatest cartilage loss was seen in the posterior subregions (> 90°). In contrast, Wirth et al^52^. reported the greatest change in the 30–75° regions. These reported ranges of regions were subgrouped into posterior regions in this study. It can be speculated that the absence of the meniscal function and subregional mechanical loading conditions may have affected the regional differences in cartilage regeneration. Yet, further research on cartilage regeneration and meniscal function is warranted.

As an interpretation of this study’s results, in the case of cartilage defects involving the anterior part of the MFC, it can be expected that good cartilage regeneration can be seen when the cartilage procedure is performed. In addition, if these defects are large in size, we can provide guidelines that hUCB-MSCs can be a good surgical option.

This study had some limitations. First, this was a retrospective study with a relatively small number of included patients in both groups. Small sample sizes may have had less power to detect statistical significances. Therefore, there is a need for well-designed prospective randomized trials with large sample sizes. Second, histological assessments, which can provide information on tissue quality of the regenerated cartilage, were not performed. Histologic assessments are currently in progress and will be evaluated in future studies. Third, there are differences in the indications for allogenic hUCB-MSC implantation and microdrilling. Marrow stimulation techniques such as MFx and microdrilling are not generally considered the standard option for the restoration of large, full-thickness cartilage defects, particularly in older patients. However, our term 'microdrilling' represents a next generation marrow stimulation technique, characterized by smaller diameter, deeper, and more numerous drillings following meticulous cartilage defect preparation^53^. This technique has shown remarkable cartilage regeneration in various pre-clinical studies. Nevertheless, it is essential to note that marrow stimulation techniques are typically more suitable for the focal cartilage defects^54,55^. We compared the two groups without considering the differences in these indications. Fourth, given that the patient group consists of OA patients, Knee injury and Osteoarthritis Outcome Scores or Western Ontario and McMaster University Osteoarthritis Index would be more suitable for evaluating the clinical outcome in this cohort than Lysholm or IKDC.

Despite these limitations, the present study had several strengths. To the best of our knowledge, this is the first study to compare the clinical, MRI, and second-look assessment outcomes using hUCB-MSCs or microdrilling for cartilage repair. We also compared the results of location-based cartilage repair regardless of the treatment method. This can aid surgeons in determining good candidates for cartilage repair procedures based on the location of the lesion in the patient.

## Conclusion

Both microdrilling and hUCB-MSC implantation combined with HTO are effective treatments for medial OA in terms of radiologic and clinical outcomes. However, hUCB-MSCs implantation was more effective than microdrilling for patient-reported outcomes and articular cartilage regeneration. In addition, the anterior lesion of the medial femoral condyle showed relatively better cartilage regeneration than lesions of other locations in both groups.

## Data Availability

The datasets generated and analyzed in the current study are not publicly available to protect the patients’ personal information but are available from the corresponding author on reasonable request.
