# Publisher Correction: Allogeneic umbilical cord blood-derived mesenchymal stem cell implantation versus microdrilling combined with high tibial osteotomy for cartilage regeneration

**DOI:** 10.1038/s41598-024-55893-x

**Published:** 2024-03-01

**Authors:** Se-Han Jung, Bum-Joon Nam, Chong-Hyuk Choi, Sungjun Kim, Min Jung, Kwangho Chung, Jisoo Park, Youngsu Jung, Sung-Hwan Kim

**Affiliations:** 1 Arthroscopy and Joint Research Institute, Yonsei University College of Medicine, Seoul, Republic of Korea; 2 Department of Orthopedic Surgery, Gangnam Severance Hospital, Yonsei University College of Medicine, 211 Eonju-ro, Gangnam-gu, Seoul, 06273 Republic of Korea; 3 Department of Orthopedic Surgery, Severance Hospital, Yonsei University College of Medicine, Seoul, Republic of Korea; 4 Department of Radiology, Gangnam Severance Hospital, Yonsei University College of Medicine, Seoul, Republic of Korea; 5 Department of Orthopedic Surgery, Yongin Severance Hospital, Yonsei University College of Medicine, Yongin, Republic of Korea

Correction to: *Scientific Reports* 10.1038/s41598-024-53598-9, published online 09 February 2024

The original version of this Article contained an error due to a system malfunction, where some sentences were partially duplicated.

As a result, in the Methods section, under the subheading ‘Surgical techniques and postoperative management’,

“After the procedure, knee motion was restricted using a hinged knee brace during daily activities for a total of 10 weeks. Continuous passive range of motion exercises were recommended immediately after surgery, starting at 60 degrees and increasing by 30 degrees every two weeks, with the goal of achieving 120 degrees to full range of motion by six weeks postoperatively. Weight-bearing was restricted for a total of 10 weeks using crutches. Toe-touch weight-bearing was permitted for the initial four weeks, followed by six weeks of partial weight-bearing, allowing for approximately 50% of the normal load during walking. After the procedure, the knee was immobilized using an extension knee splint for 2 weeks. Continuous passive range of motion exercise was recommended from 2 weeks after surgery, and weight-bearing was restricted for 10 weeks. Toe-touch weight-bearing was allowed for 4 weeks, following which partial weight-bearing was allowed for 6 weeks.”

now reads:

“After the procedure, knee motion was restricted using a hinged knee brace during daily activities for a total of 10 weeks. Continuous passive range of motion exercises were recommended immediately after surgery, starting at 60 degrees and increasing by 30 degrees every two weeks, with the goal of achieving 120 degrees to full range of motion by six weeks postoperatively. Weight-bearing was restricted for a total of 10 weeks using crutches. Toe-touch weight-bearing was permitted for the initial four weeks, followed by six weeks of partial weight-bearing, allowing for approximately 50% of the normal load during walking.”

The original Article has been corrected.

